# Proteomic Analyses of Mammary Glands Provide Insight into the Immunity and Metabolism Pathways Associated with Clinical Mastitis in Meat Sheep

**DOI:** 10.3390/ani9060309

**Published:** 2019-05-31

**Authors:** Jianfeng Gao, Taotao Li, Zengkui Lu, Xia Wang, Xingxu Zhao, Youji Ma

**Affiliations:** 1 College of Animal Science and Technology, Gansu Agricultural University, Lanzhou 730070, China; jfgao1992@163.com (J.G.); ttli2018@163.com (T.L.); luck@st.gsau.edu.cn (Z.L.); wangxiaandzisu@163.com (X.W.); 2 Sheep Breeding Biotechnology Engineering Laboratory of Gansu Province, Minqin 733300, China; 3 College of Veterinary Medicine, Gansu Agricultural University, Lanzhou 730070, China; zhaoxx@gsau.edu.cn

**Keywords:** sheep, mastitis, mammary gland, proteomics, two-dimensional electrophoresis, immune response

## Abstract

**Simple Summary:**

Clinical mastitis is one of the most common diseases in sheep and is of major economic concern due to treatment costs, inadequate lamb growth and premature eliminate of ewes. To preliminarily explore possible regulatory roles of proteins involved in the host-pathogen interactions during intramammary infection triggered by this disease in meat sheep, mammary tissues were harvested from sheep with healthy and clinical mastitis caused by natural infection, and the differentially expressed proteins were identified in an infected group when compared to a healthy group, using comparative proteomics based on two-dimensional electrophoresis. Further enrichment analyses indicated that most of the differentially expressed proteins mainly engaged in regulating immune responses and metabolisms. These findings offer candidate proteins for further studies on molecular mechanisms of host defense response and metabolism in sheep cases.

**Abstract:**

Clinical mastitis is still an intractable problem for sheep breeding. The natural immunologic mechanisms of the mammary gland against infections are not yet understood. For a better understanding of the disease-associated proteins during clinical mastitis in meat sheep, we performed two-dimensional electrophoresis (2-DE)-based comparative proteomic analyses of mammary tissues, including from healthy mammary tissues (HMTs) and from mammary tissues with clinical mastitis (CMMTs). The 2-DE results showed that a total of 10 up-regulated and 16 down-regulated proteins were identified in CMMTs when compared to HMTs. Of these, Gene Ontology (GO) and Kyoto Encyclopaedia of Genes and Genomes (KEGG) enrichment analyses revealed that most proteins were associated with immune responses or metabolisms. The results of qRT-PCR and Western blot for randomly selected four differentially expressed proteins (DEPs) including superoxide dismutase [Mn] (SOD2), annexin A2 (ANAX2), keratin 10 (KRT10) and endoplasmic reticulum resident protein 29 (ERP29) showed that their expression trends were consistent with 2-DE results except *ANXA2* mRNA levels. This is an initial report describing the 2-DE-based proteomics study of the meat sheep mammary gland with clinical mastitis caused by natural infection, which provides additional insight into the immune and metabolic mechanisms during sheep mastitis.

## 1. Introduction

Mastitis, or inflammation of the mammary gland, is a serious concern in mammals, particularly cattle and sheep, which is mainly caused by bacterial infections [1,2]. In sheep flocks, mastitis usually results in great economic losses for sheep breeding through loss of milk yield and quality, low weight of weaning lambs, and death or early culling of lambs and infected ewes [3]. Moreover, clinical mastitis is also of great welfare concern due to swelling and pain in the affected udder [4]. Clinical mastitis is defined as a series of common clinical symptoms of udders including swelling, warmth, pain and discomfort, as well as changes in milk composition and appearance [1,5,6,7]. Over the last few decades, relevant studies on mastitis in sheep mainly focus on bacteriology [1,8], epidemiology [6,8], diagnosis [1,3] and preventive and therapeutic measures [1,7]. These practices have hindered the development of the disease; however, a safe and effective prevention program has yet to be found and formulated, owing to the complex causes of mastitis.

Fortunately, with the development and application in the field of life science of modern molecular biology and biological technology, especially genomics, transcriptomics and proteomics, they offer hope for the identification of associated genes and potential molecular targets during mastitis. In particular, proteomics has been widely used to investigate proteome changes and excavate potential protein targets from the mammary gland [9,10], milk [11,12], and blood [13,14] during mastitis in cattle, and less research in dairy sheep [15], but no report in meat sheep. In addition, previous studies on proteomics in the mammary gland with clinical mastitis are mainly focused on mastitis caused by experimental infection with single bacterium [9,10,16], but proteomic information on the mammary gland with this clinical case caused by natural infection is still poorly understood. This work was therefore carried out to identify the differentially expressed proteins (DEPs) in mammary tissues with clinical mastitis (CMMTs) and healthy mammary tissues (HMTs) from meat sheep and to screen the disease-related proteins using two-dimensional electrophoresis (2-DE) combined with mass spectrometry.

## 2. Materials and Methods

### 2.1. Animals and Tissue Collection

All experimental work was performed according to the Regulation on the Administration of Experimental Animals published by the Ministry of Science and Technology of the People’s Republic of China in 2006 (Approval No. 2006-398) and was approved by the Animal Care Committee of Gansu Agricultural University (Approval No. GSAU-AEW-2017-0003). Six multiparous (second lactation) purebred female Hu sheep at the same age (2.1 years old) and similar weight (51.4–53.1 kg), with half healthy and half infection with clinical mastitis, were provided by the Pingchang Modern Agriculture and Animal Husbandry Company-Hu Sheep Breeding Base (Lintao, China). All experimental animals were housed under similar conditions of free access to food (total mixed ration) and water in natural lighting. We performed the examination of clinical characteristics and pathogens for all experimental animals to ascertain whether they were healthy or clinical mastitis symptoms. Clinical diagnosis showed three infected ewes were characterized by swelling, redness, and necrosis of one or more half-udders, along with the abnormal discharge of milk (the presence of clots or serum). For pathogen examination, 200 μL of milk samples from each udder quarter were collected under sterile conditions and spread on a MacConkey Agar medium plate (Huankai Microbial, Guangzhou, China), Baird-Parker medium plate (Huankai Microbial, Guangzhou, China) and 5% sheep blood agar plate (Huankai Microbial, Guangzhou, China), respectively, followed by culture for 18–36 h at 37 °C. The colonial morphology, Gram-staining characteristics and biochemical features from isolates were assessed. The microbiological examination suggested all three clinical cases used in this study were caused by mixed infections with *Staphylococcus aureus* and *Escherichia coli*. After sheep were slaughtered, mammary tissue samples from each ewe were harvested under sterile conditions and washed with PBS to remove blood and milk from tissue samples, then cryopreserved immediately in liquid nitrogen. Parts of each mammary tissue were fixed in 4% paraformaldehyde solution (Solarbio, Beijing, China) for 48 h, dehydrated, cleared, and embedded in paraffin.

### 2.2. H&E Staining and Masson Staining

Paraffin sections (4 μm) were prepared for H&E and Masson staining. The histomorphological changes in the mammary glands were observed under the optical microscope (Sunny EX31, Ningbo, China). The images for sections were acquired by MvImage software (Sunny, Ningbo, China). For H&E staining, sections from HMTs and CMMTs groups were stained with hematoxylin and eosin, dehydrated, dewaxed with conventional histological methods as described by Hara et al. [17] with some modifications.

### 2.3. Protein Extraction

Total protein was extracted from mammary tissues using 10% trichloroacetic acid (TCA) (Beyotime, Beijing, China) (prepared with acetone, containing l g of TCA and 0.07% β-mercaptoethanol). And then, 1 mL of the liquid mixture was added to a centrifuge tube, shaken for 5 min, kept overnight, and centrifuged at 12,000 *g*, 4 °C for 40 min. The supernatant was discarded and washed with cold acetone, and then centrifuged at 12,000 *g*, 4 °C for 10 min. After the supernatant was discarded, the steps were repeated three times. The pellet was dried and then dissolved in lysis buffer containing 7 M urea (Bio-Rad, Hercules, CA, USA), 2 M thiourea (Bio-Rad, Hercules, CA, USA), 4% 3-[(3-cholamidopropyl)dimethylammonio]-1-propanesulfonate (CHAPS) (Bio-Rad, Hercules, CA, USA), 40 mM dithiothreitol (DTT) (Solarbio, Beijing, China), 40 mM Tris (Solarbio, Beijing, China)and 2% immobilized pH gradients (IPG) buffer (ReadyStripTM IPG strips, Bio-Rad, Hercules, CA, USA). After centrifugation at 1000 g, 4 °C for 10 min, the supernatant was collected, quantified, and then stored at −80 °C for 2-DE analysis.

### 2.4. 2-DE and Image Analysis

Protein samples were subjected to 2-DE using immobilized pH gradient (IPG) strips (17 cm, pH 3–10, Bio-Rad) in the first dimension and discontinuous 12% sodium dodecyl sulphate-polyacrylamide electrophoresis (SDS-PAGE) gels in the second dimension, as described previously [18]. The polyacrylamide gels were stained with colloidal Coomassie G250 overnight, and the images of three gels for each sample were captured using a Powerlook 2100XL-USB image scanner (UMAX, Taipei, Taiwan). The protein spots in gel images were matched and analyzed using PDquest 8.0 software package (Bio-Rad, Hercules, CA, USA). Before automatically matching protein spots, background of the gel images was removed as described previously [18]. The relative volume of protein spot was calculated and considered as its expression level. We selected the spots with statistically significant changes at protein level in all replicate gels from HMTs and CMMTs groups by independent *t*-test, and then used for further analysis 

### 2.5. In-Gel Digestion and MALDI-TOF Analysis

Each of the selected differential protein spots was excised manually by using pipette tips and subsequently de-stained with 100 mM NH_4_HCO_3_ in 30% acetonitrile solution (ACN, Solarbio, Beijing, China). After being aspirated and freeze-dried, the samples were digested with 5 μL of 2.5–10 ng/μL sequencing-grade modified trypsin (Promega, Madison, WI, USA) overnight at 37 °C. The Peptides were extracted three times with 60% ACN and 0.1% trifluoroacetic acid (TFA, Solarbio, Beijing, China), freeze-dried and then resuspended in 5 mg/mL cyano-4-hydroxycinnamic acid (Bio-Rad) in 50% ACN and 0.1% TFA for matrix-assisted laser desorption/ionization time of flight-mass spectrometry (MALDI TOF-MS) analysis. Peptide mass was determined using MALDI TOF-MS on a 4800 Plus MALDI TOF/TOF™ Analyzer (Applied Biosystems, Massachusetts, Framingham, MA, USA). Peptide mass maps were acquired in a positive ion reflector mode (2 kV accelerating voltage) with 355-nm laser shots for per spectrum. The minimum signal-to-noise ratio was set at 50. MS spectra for all samples were measured with an overall mass/charge (m/z) range of 800 to 4000, and eight of the most intense ions were selected as precursors for the tandem mass spectrometry (MS/MS) acquisition.

### 2.6. Protein Identification and Functional Enrichment Analysis

Proteins were identified using Mascot peptide mass fingerprinting software 2.2 (Matrix Science, Boston, MA, USA) to search against the NCBI non-redundant protein database for *Ovis aries*, the Uniprot protein database and Swiss-Prot database. The functional annotation and pathway enrichment of DEPs were performed using DAVID (SAIC-Frederick Inc, Frederick, MD, USA)and Cytoscape online software (https://cytoscape.org/). The protein-protein interaction (PPI) network was visualized using STRING Version 11.0 (http://string-db.org).

### 2.7. Total RNA Isolation and cDNA Synthesis

Total RNA from frozen HMTs and CMMTs was isolated with a TRIzol reagent (TransGen, Beijing, China). The concentration and purity of RNA were determined at A260, 280, and A230 using NanoVue Plus, and RNA integrity was evaluated by separating 1 μg total RNA on 1.5% agarose gel containing 1% formaldehyde. Equal amounts of RNA samples (250 ng) were reverse transcribed to cDNA following instructions of the Revert Aid First Strand cDNA Synthesis Kit (TransGen, Beijing, China) and then stored at −20 °C until use.

### 2.8. Quantitative Real-Time PCR (qRT-PCR) Analysis

The mRNA expression profiles of randomly selected four DEPs, including ANXA2 (immunity-related), SOD2 (metabolism-related), KRT10 (cell proliferation-related) and ERP29 (cell apoptosis-related) were measured by qRT-PCR with *GAPDH* gene as an internal control. The primer pairs were designed using Primer Premier 5.0 software (Table 1) and then synthesized by Sagan Biotech (Shanghai, China). The qRT-PCR was carried out using a LightCycler^®^ 480 qRT-PCR system (Roche, Basel, Switzerland) and *TransStart*^®^ Tip Green qPCR SuperMix Kit (TransGen, Beijing, China) following the manufacturer’s instructions. The cycling conditions were as follows: 94 °C for 5 s, 40 cycles of 94 °C for 30 s, an annealing step at 56 °C for 15 s, followed by 10 s at 72 °C for final extensions. The qRT-PCR reaction was performed in a volume of 20 μL, which included 1 μL of cDNA, 10 μL of 2×*TransStart*^®^ Tip Green qPCR SuperMix, 0.4 μL of Passive Reference Dye (50×), 7.8 μL of ultra-purified water and 0.4 μL each of the forward and reverse primers (10 μmol/L). Relative mRNA expressions of target genes were calculated using the 2^−ΔΔCt^ method [19].

### 2.9. Western Blot

Total protein was extracted from frozen HMTs and CMMTs using radio immunoprecipitation assay (RIPA) kit (Solarbio, Beijing, China) and quantified using a BCA Protein Assay kit (Beyotime, Shanghai, China). Protein was denatured and then stored at −20 °C. Equal amounts of protein (30 μg) were loaded onto 12% polyacrylamide gels and separated by sodium dodecyl sulfate-polyacrylamide gel electrophoresis (SDS-PAGE). Gels were then transferred to PVDF membrane for Western blot. Membranes were blocked by 5% (g/mL) skimmed milk powder and were subsequently incubated with the following primary antibodies: mouse anti-glyceraldehyde-3-phosphate dehydrogenase (GAPDH) (1:2000; Bioss, Beijing, China), rabbit anti- superoxide dismutase [Mn](SOD2) (1:500; Bioss, Beijing, China), rabbit anti-annexin A2 (ANXA2) (1:500; Bioss, Beijing, China), rabbit anti- endoplasmic reticulum resident protein 29 (ERp29) (1:500; Bioss, Beijing, China), or rabbit anti- keratin 10 (KRT10) (1:500; Bioss, Beijing, China) overnight at 4 °C. Blots were incubated with the goat anti-mouse IgG/HRP (horse-radish peroxidase) secondary antibody (1:5000; Bioss, Beijing, China) or goat anti-rabbit IgG/HRP secondary antibody (1:5000; Bioss, Beijing, China) at 37 °C for 1.5 h. Immunoreactive bands of each protein were visualized using an enhanced chemiluminescence (ECL) kit (NCM Biotech, Suzhou, China), developer and fixer (Kodak, Rochester, NY, USA) in an X-ray room.

### 2.10. Statistical Analysis

Statistical analysis of all data was performed using SPSS 19.0 (SPSS, Chicago, IL, USA), and differences between the two groups were assessed by the Independent Samples *t*-test. The results were presented in the form of bar charts as mean ± SD. Values of *p* < 0.05 were considered statistically significant.

## 3. Results

### 3.1. Morphological Comparisons of Mammary Tissues with Healthy and Clinical Mastitis

**Figure 1:**
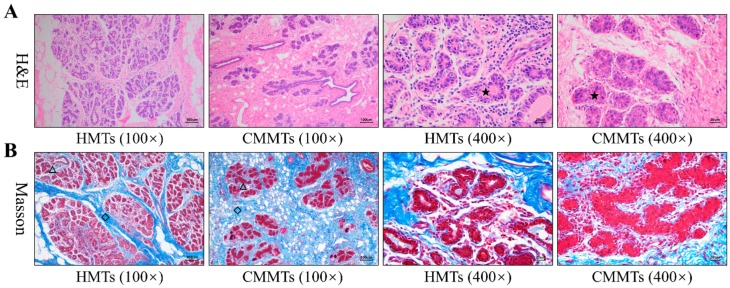
Morphological comparisons of mammary tissues from HMTs and CMMTs disease symptoms. (**A**) H&E staining; (**B**) Masson staining. The pentagram denotes mammary acinus, diamond denotes interlobular connective tissues, and triangle denotes intralobular connective tissues. HMTs, healthy mammary tissues. CMMTs, mammary tissues with clinical mastitis.

H&E staining showed that mammary epithelial cells were arranged in good order, and milk was observed in lumen of acinus from HMTs group, while epithelial cells were loss and arranged irregularly, and a large number of inflammatory cells infiltrated in atrophic acinar lumen with cellular debris from CMMTs group (Figure 1A). Masson staining revealed that hyperplasia of connective tissues, especially interlobular connective tissues were evident from CMMTs group in comparison to HMTs group (Figure 1B).

### 3.2. Distinct Protein Patterns in Mammary Glands of Healthy and Clinical Mastitis-Afflicted Sheep

**Figure 2:**
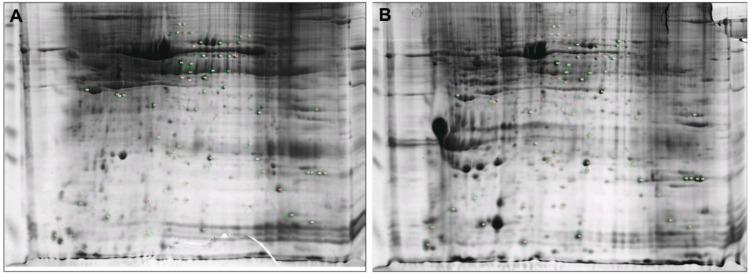
Representative 2-DE gels of mammary tissues from healthy mammary tissues (HMTs) and mammary tissues with clinical mastitis (CMMTs) groups. (**A**) HMTs group; (**B**) CMMTs group. 600 μg proteins were separated on pH 3–10 IPG nonlinear strips and on 12% sodium dodecyl sulfate-polyacrylamide gel electrophoresis (SDS-PAGE).

The 2-DE proteomic profiles of HMTs and CMMTs were obtained using the TCA/acetone precipitation method with 600 μg of sample volume, a 90,000 Vh focusing time, a 12% gel and pH 3–10 test strips. The representative 2-DE gel images for HMTs and CMMTs groups were shown in Figure 2, showing excellent protein resolution.

### 3.3. Identification of Differentially Expressed Proteins Using MALDI-TOF/TOF-MS

Differentially expressed proteins (n = 26) in abundance were successfully identified by MALDI-TOF/TOF-MS in CMMTs group as compared to HMTs, based on abundance variation of threshold |fold change| >1.5 and *p* < 0.05. Of these, 10 proteins were up-regulated, and 16 were down-regulated in CMMTs as enlisted in Table 2.

### 3.4. Functional Annotation and Pathway Enrichment Analysis of DEPs

**Figure 3:**
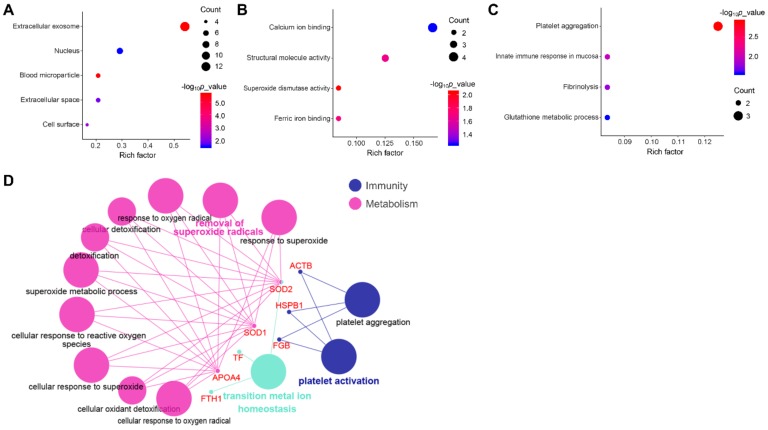
Gene Ontology (GO) annotation of DEPs. GO enrichment analyses of differentially expressed proteins involved in (**A**) cellular component; (**B**) molecular function, and (**C**) biological process from the Database for Annotation, Visualization and Integrated Discovery (DAVID) database; (**D**) GO analyses of DEPs involved in biological processes using Cytoscape software.

The results of GO analyses from three main categories, namely cell component, molecular function and biological process were provided in Figure 3. Among cell components, most DEPs were involved in extracellular and nuclear components (Figure 3A). Among molecular functions, DEPs were mainly engaged in binding, structural molecule activity and superoxide dismutase activity (Figure 3B). Among biological processes, DEPs were primarily concentrated on innate immune response, platelet activation and aggregation, and metabolic process (Figure 3C,D).

**Figure 4:**
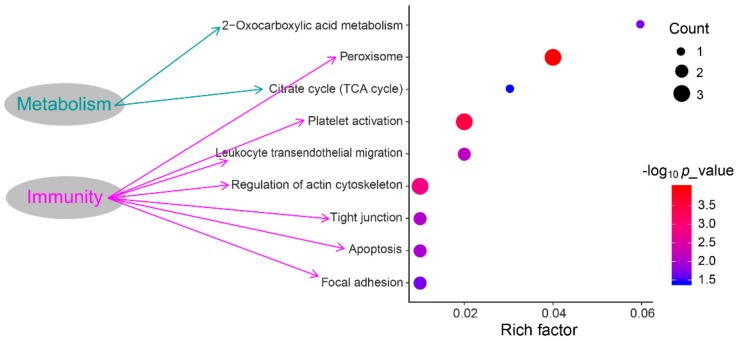
The Kyoto Encyclopedia of Genes and Genomes (KEGG) enrichment pathways of DEPs.

The KEGG pathway analyses revealed that DEPS were significantly enriched in 18 KEGG pathways. Of these, seven pathways were associated with immune responses such as platelet activation, leukocyte transendothelial migration, etc, as well as two pathways associated with metabolisms including peroxisome and 2-Oxocarboxylic acid metabolism (Figure 4).

### 3.5. PPI Network Analysis

**Figure 5:**
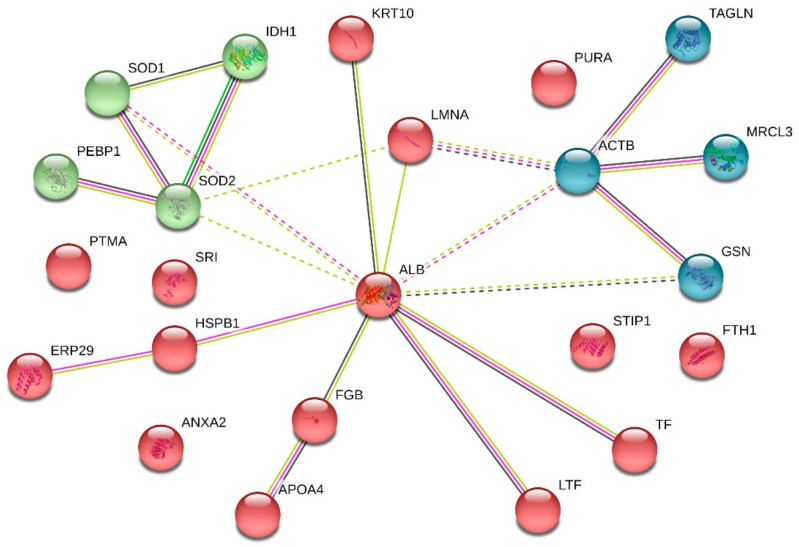
PPI network analysis of DEPs in CMMTs. The nodes represent proteins, and the edges represent the predicted functional associations. The green line represents neighboring evidence, the purple line represents experimental evidence, the yellow line represents textual evidence, and the black line represents co-expressive evidence. CMMTs, mammary tissues with clinical mastitis. DEPs, differentially expressed proteins.

The PPI network of DEPs in CMMTs was constructed using STRING software. Seventeen of these 26 DEPs were associated functionally with at least one other protein, as shown in Figure 5. Lines with different colors between proteins show the different types of interaction evidence. According to information in the STRING database (https://string-db.org/cgi/input.pl), the PPI networks of the DEPs contained 17 nodes, 21 edges and 50 connections. In the PPI network, the top five high-degree hub nodes included serum albumin (ALB), actin, cytoplasmic 1 (ACTB), superoxide dismutase [Mn] (SOD2), superoxide dismutase [Cu-Zn] (SOD1) and isocitrate dehydrogenase 1 (IDH1).

### 3.6. Verification of Differentially Expressed of Proteins

**Figure 6:**
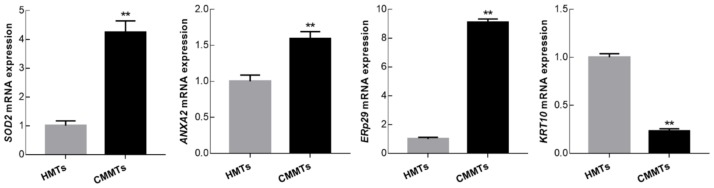
Relative mRNA expressions for selected randomly four differentially expressed proteins (DEPs) were detected by qRT-PCR. Data show means ± SD (n = 3). ** *p* < 0.01. HMTs, healthy mammary tissues. CMMTs, mammary tissues with clinical mastitis.

**Figure 7:**
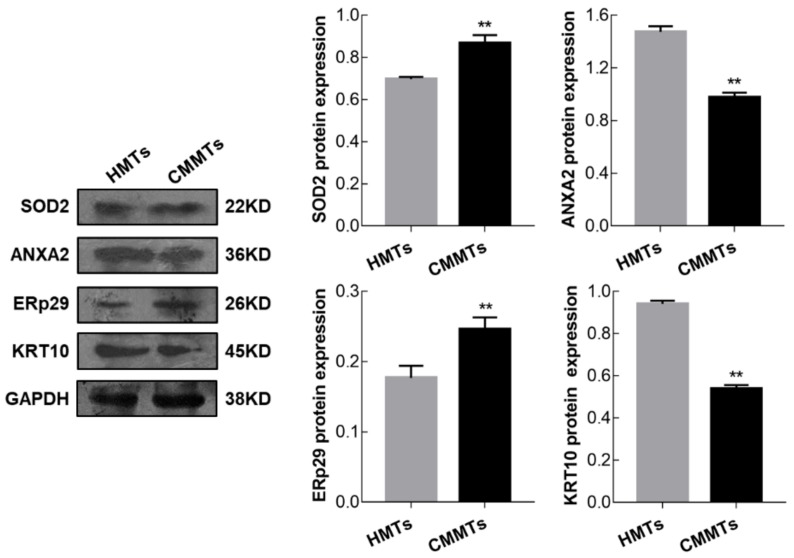
Expressions for selected randomly four DEPs were detected by Western blot. Data show means ± SD (n = 3). ** *p* < 0.01. HMTs, healthy mammary tissues. CMMTs, mammary tissues with clinical mastitis. DEPs, differentially expressed proteins.

To confirm the accuracy of the 2-DE results, the expression levels from four DEPs including SOD2, annexin A2 (ANXA2), endoplasmic reticulum protein 29 (ERp29), and keratin 10 (KRT10) were examined at mRNA and protein levels by qRT-PCR and Western blotting, respectively. qRT-PCR results showed that relative mRNA abundance of *SOD2*, *ANXA2* and *ERp29* was significantly higher, whereas *KRT10* mRNA relative abundance was significantly lower in CMMTs group as compared to HMTs group (*t*-test, *p* < 0.01) (Figure 6). Western blot indicated that expressions of SOD2 and ERp29 proteins were significantly up-regulated, whereas ANXA2 and KRT10 proteins were significantly down-regulated in CMMTs group in comparison with HMTs group (*t*-test, *p* < 0.01) (Figure 7).

## 4. Discussion

Chinese Hu sheep, a meat-produced sheep breed, is renowned for its high fecundity and excellent milk production [20]. It is an ideal resource for researching the molecular pathogenesis of sheep clinical mastitis. Morphologically, our observations revealed that mammary glands from CMMTs group exhibited obvious hyperplasia of inter-lobular connective tissues in the stromal regions and infiltration of inflammatory cells into the atrophic lumen of alveoli as compared to HMTs group, which were similar to the reports of Gong et al. [21] in murine mastitis. These suggest that histological structures of mammary glands with mastitis have changed obviously, then result in the changes of mammary microenvironment and biological function.

This is the first study to estimate protein changes in CMMTs and HMTs from meat-producing sheep flocks by using 2-DE, thereby exploiting protein targets associated with the occurrence and development of sheep clinical mastitis. As one of the most complex and devastating diseases in sheep breeding, mastitis significantly reduces production performance [6,22]. The host response to pathogens in the occurrence and development of mastitis was controlled by many genes whose expression confirmed at the levels of transcription and translation [10]. In this study, we successfully separated and obtained the protein profiles of CMMTs and HMTs. A total of 26 DEPs were successfully identified in CMMTs as compared to HMTs. Of the 26 DEPs, 10 proteins were up-regulated, and 16 proteins were down-regulated. All DEPs were subsequently subjected to GO annotation and KEGG enrichment analysis. According to functional classification and enrichment, most DEPs were primarily involved with the metabolism like apolipoprotein A4 (APOA4), SOD1, SOD2 and IDH1, and immune response including those associated with mucosal innate immunity, leukocyte transendothelial migration, platelet activation and aggregation, and its relation with proteins such as APOA4, lactotransferrin (LTF), fibrinogen beta chain (FGB), myosin regulatory light chain MRCL3 (MRCL3), cofilin 1 (CFL1) and gelsolin (GSN). Several studies of this disease have indicated that infected mammary gland tissue was found to significantly up-regulate expression of genes related to the immune and inflammatory responses and down-regulate genes related to metabolism processes such as lipid metabolism, fat metabolism, etc. [23,24]. During mastitis, invasive pathogenic bacteria activates the immune response systems, such as innate immunity [25], cellular immunity [26,27] and humoral immunity [26]. The mucosal immune system is the main site of local non-specific immune function and plays important roles in function of the innate immune system, serving as the first-line of defence against infections [28], which consists of intestinal, respiratory, urogenital mucosa, and the exocrine glands [26]. Mammary glands are one of the key components for the integrated mucosal immune system to prevent causative pathogens from host cells as a selective barrier against invading pathogens during intramammary infection [29]. Our results indicated that two DEPs (APOA4 and LTF) participated in innate immune response in mucosa considering the biological process category. 

APOA4, a member of the apolipoprotein family, is implicated in immune response [30] and lipid metabolism [31]. As an immunomodulatory factor, it has been reported that APOA4 plays an important role at the site of primary immune responses in the small intestine [30]. A previous proteome study on healthy sheep milk documented that APOA4 involved in two biological processes including innate immune response and host defense response [32]. Our results from 2-DE analyses showed APOA4 protein expression was reduced in CMMTs compared to healthy controls, but the expression patterns on APOA4 in mastitic mammary tissues from sheep and other mammals have not been found in literature. Therefore, whether APOA4 plays an important role in regulating immune response during sheep mastitis, which remains to be fully elucidated.

LTF, a potential indicator of mastitis in dairy cows, is an iron-binding glycoprotein found in milk, which is mainly released by the mammary epithelial cells with close associations to the immune systems [33,34]. Moreover, LTF can also be synthesized and secreted by polymorphonuclear neutrophils during an inflammatory response [35]. In bovine mastitis, LTF contributes to innate immunity to pathogenic bacteria by limiting microbial access to iron [36]. In addition, previous studies showed that the polymorphisms present in the bovine *LTF* gene are closely connected to mastitis resistance or susceptibility [34,36]. LTF is a low level of expression in the milk [35] and mammary glands [37,38] from healthy cows, but it is dramatically increased in cows with clinical mastitis. Similarly, we also observed that LTF protein was up-regulated in CMMTs group. It can be speculated that the *LTF* gene plays a key role in sheep mastitis to prevent the invasion of pathogen infection. However, whether ovine mastitis is associated with the polymorphisms existing in the *LTF* gene, which still needs to be further investigated.

In this work, GO analysis using Cytoscape software showed that up-regulated proteins in CMMTs group, SOD1 and SOD2, were significantly involved in the metabolic response to reactive oxygen species (ROS). Under normal physiological conditions, ROS participate in redox reactions and serve as second messengers for regulatory functions [39]. However, infected tissues and cells accumulate excessive ROS owing to metabolic abnormalities, which needs an improved antioxidant system to prevent cellular damage [39]. SOD1 and SOD2, as two isoforms of superoxide dismutases in mammals, are antioxidative enzymes that catalyze the degradation and removal of ROS [40]. In this study, we found the expression of SOD2 at mRNA and protein levels were up-regulated in the mammary glands of ewes with clinical mastitis compared to healthy ewes. Results are consistent with estimates of Mitterhuemer et al. [37], which report *SOD2* gene level is evidently increasing in mammary tissue from mastitis cows inoculated with *E. coli* 24 h after infection as compared to controls. Moreover, Kosciuczuk et al. [38] also reported that *SOD2* mRNA expression is significantly up-regulated in mammary glands from the first or second lactation cows infected with *Staphylococci* mastitis, compared to healthy ones. In this study, another homologous protein SOD1 with high expression was also discovered in sheep mammary glands with clinical mastitis. Results suggested that these proteins play a role in the occurrence and development of mastitis, but a specific regulatory mechanism still merits further study.

IDH1, an important metabolic enzyme, has been previously documented that it is involved in lipid metabolism and milk fat synthesis during bovine mammary gland development [41]. Additionally, IDH1 is implicated in immune responses in various diseases, and its expression down-regulation or even mutation results in immune inhibition [42,43]. Our 2-DE results showed that IDH1 was down-regulated significantly in CMMTs group, which is in accord with estimates in mammary tissues from dairy sheep with clinical mastitis reported by Banos et al. [43]. These findings illustrate it is likely to be involved in protective immunity or milk fat biosynthesis in the mammary gland of sheep flocks infected with clinical mastitis.

ANXA2, a member of the multigene family of annexin proteins, exists in many cells and is engaged in several biological processes, such as immune responses [44], anti-inflammatory effects [45], cell proliferation and apoptosis [46]. It has been reported that *Anxa2*-deficient mice exhibited enhanced inflammatory responses during fungal infection [47]. In this study, lower ANXA2 protein abundance was examined in CMMTs when compared to HMTs. Conversely, *ANXA2* mRNA abundance was up-regulated in CMMTs group, which is in accordance with previous studies on cow mastitis, including from mammary glands infected with *Escherichia coli* mastitis reported by Mitterhuemer et al. [37], and from mammary glands infected with coagulase-negative *Staphylococci* mastitis reported by Kosciuczuk et al. [38]. The expression differences at mRNA and protein levels may be caused by post-transcriptional regulation (such as mRNA stability) [48,49] and posttranslational protein modifications (such as protein stability and phosphorylation) [50]. In bovine mastitis, Zhang et al. [10] reported that the expression patterns of several genes at mRNA level were in opposition to those at protein expression. For ANXA2, previous studies documented that its expression was subjected to various post-translational regulation, such as phosphorylation and ubiquitination [51]. These implicate a potential role for ANXA2 in immunity and inhibition of inflammation during mastitis, but the specific regulatory role still needs further exploration.

KRT10 is a member of the cytokeratin family of type I. Keratins are a highly diverse family of cytoskeletal fibrous proteins that are important markers for epithelial cell differentiation, and can be divided into two categories: type I (acidic keratins) and type II (basic-to-neutral keratins) [52]. Cytokeratin not only plays a role in maintaining the integrity of epithelial cells and tissues, but it also participates in several processes including cell signal transduction and mitosis [53] in addition to various other stress responses [54]. Studies have shown that KRT10 protein has differential expression profiles in tissues or cells, including lung and colorectal cancers, epidermolysis palmoplantar keratoderma lesions, and coronary heart disease. In the present study, decreased KRT10 mRNA and protein levels were observed in CMMTs relative to HMTs. It is reported that keratins can adhere pathogens and inhibit its activity, thus preventing pathogenic microorganisms from moving and entering mammary glands [55,56]. For instance, Smolenski et al. [56] documented that multiple members of the keratin family, including KRT1, KRT4, KRT14 and KRT17, as well as protein KRT10, are involved in immune defense against pathogens in infected bovine mammary glands. Therefore, the downed-regulated of *KRT10* gene expression is likely related to the occurrence and development of ovine mastitis.

## 5. Conclusions

This is the first report describing the proteome changes in mammary glands from meat sheep with clinical mastitis caused by natural infection. In this work, we identified 10 up-regulated and 16 down-regulated proteins in CMMTs in comparison with HMTs using 2-DE analysis and found that most of these DEPs were closely related to immune responses and metabolisms. Our findings will enhance the knowledge of proteins with naturally infected sheep clinical mastitis, and will also contribute to research of molecular mechanisms on host defense and metabolism during sheep mastitis.

## Figures and Tables

**Table 1:** Primers of qRT–PCR used in this study.

Gene	Primer Sequences	Accession No.	Products Length (bp)
*SOD2*	F: TAAACCGTCAGCCTTACACC	NM_001280703.1	198
	R: ACATTTTCAAACAGTTGCCTA		
*ANXA2*	F: CAAGCCCCTGTATTTCGCTGA	NM_001093788.1	194
	R: CTTTCTGGTAGTCGCCCTT		
*ERp29*	F: CCTTCCCCTGGATACAATCACT	EU596595.1	125
	R: AGTTTTCAGCCAGACGCTTG		
*KRT10*	F: TGCCCCAGGTGTTGATCTCACT	XM_015098774.1	100
	R: ATTGAACCATGCTTCGGCGTCT		
*GAPDH*	F: GAAGGTCGGAGTGAACGGAT	NM_001190390.1	196
	R: GATGACGAGCTTCCCGTTCT		

**Table 2:** Differentially expressed proteins were identified by matrix assisted laser desorption ionization-time of flight mass spectrometry (MALDI-TOF-TOFMS) in mammary tissues with clinical mastitis (CMMTs) as compared with healthy mammary tissues (HMTs).

Spot No.	Protein Name	Gene Name	Fold Change	MW(Da)	pI
791	Lactoferrin	LTF	1.78	77186.4	8.4
1040	Lamin A/C	LMNA	−3.12	65082.6	6.54
844	Transferrin	TF	−4.39	77290.5	6.41
1785	Annexin A2	ANXA2	−1.83	39021	7.71
2244	Keratin 10	KRT10	−4.47	57248.7	5.4
1043	STIP1	STIP1	−1.76	68120	6.81
1260	Actin, cytoplasmic 1	ACTB	−1.79	41967.9	5.29
2234	Cathelicidin	CATH1	1.58	17637	7.55
2182	PEBP1	PEBP1	2.72	21002.7	6.96
2459	Prothymosin alpha	PTMA	1.86	6228.8	4.1
1642	Apolipoprotein A4	APOA4	−1.94	41454.5	5.55
2292	Ferritin heavy chain	FTH1	−2.58	21037.2	5.53
1709	Albumin	ALB	−3.03	66269.8	5.58
2358	Beta-lactoglobulin	BLG	2.03	18139.4	5.26
741	Gelsolin	GSN	−3.08	80652.5	5.49
2190	Transgelin	TAGLN	3.19	22866.5	8.92
2252	Sorcin	SRI	−1.65	20317.8	5.11
1070	Fibrinogen beta chain	FGB	−3.15	56604.7	7.84
2446	Superoxide dismutase [Cu-Zn]	SOD1	4.85	15684.9	6.14
2364	Superoxide dismutase [Mn]	SOD2	1.85	24135.4	8.67
1560	40S ribosomal protein SA	RPSA	−1.83	33538.8	4.8
1580	Transcriptional activator protein Pur-alpha	PURA	−1.75	30258.4	7.05
2301	Myosin regulatory light chain MRCL3	MRCL3	−1.85	19794.5	4.72
2485	Heat shock protein family B member 1	HSPB1	4.74	22437.3	5.77
1971	Endoplasmic reticulum resident protein 29	ERP29	1.58	29034.1	5.64
1358	Isocitrate dehydrogenase [NADP]	IDH1	−1.97	46703.5	6.34

MW, molecular weight; pI, isoelectric point.
